# Listening to the Body: Interoceptive Awareness and Eating Disorder Vulnerability in Adolescents with Inflammatory Bowel Diseases

**DOI:** 10.3390/children13050626

**Published:** 2026-04-30

**Authors:** Anna Riva, Gabriele Arienti, Simona Di Guardo, Eleonora Brasola, Giovanna Zuin, Laura Spini, Naire Sansotta, Andrea Eugenio Cavanna, Renata Nacinovich

**Affiliations:** 1 Department of Child Neuropsychiatry, Fondazione IRCCS San Gerardo dei Tintori, 20900 Monza, Italy; anna.riva@irccs-sangerardo.it (A.R.); gabriele.arienti@irccs-sangerardo.it (G.A.); simona.diguardo@irccs-sangerardo.it (S.D.G.); eleonora.brasola@irccs-sangerardo.it (E.B.); l.spini4@campus.unimib.it (L.S.); 2 Department of Pediatrics, Fondazione IRCCS San Gerardo dei Tintori, 20900 Monza, Italy; giovanna.zuin@irccs-sangerardo.it; 3 School of Medicine and Surgery, University of Milano-Bicocca, 20900 Monza, Italy; a.e.cavanna@bham.ac.uk; 4 Department of Paediatric Hepatology Gastroenterology and Transplantation, Papa Giovanni XXIII Hospital, 24127 Bergamo, Italy; nsansotta@asst-pg23.it; 5 Department of Neuropsychiatry, National Centre for Mental Health, Birmingham and Solihull Mental Health NHS Foundation Trust, Birmingham B15 2FG, UK; 6 School of Medical Sciences, College of Medicine and Health, University of Birmingham, Birmingham B15 2TT, UK; 7 School of Life and Health Sciences, Aston Brain Centre, Aston University, Birmingham B4 7ET, UK; 8 Sobell Department of Motor Neuroscience and Movement Disorders, Institute of Neurology, University College London, London WC1N 3BG, UK

**Keywords:** interoception, inflammatory bowel diseases, eating disorders, gastrointestinal disorders, adolescents

## Abstract

**Highlights:**

**What are the main findings?**

- Approximately 15.8% of adolescents with IBDs were at high risk for developing eating disorders, a considerably higher prevalence compared to the general adolescent population.
- Adolescents with IBDs at high risk showed significantly higher EDI-3 scores and lower interoceptive trust (MAIA-2 “Trusting”), indicating greater psychological vulnerability and reduced confidence in bodily signals.

**What are the implications of the main findings?**

- Routine screening for eating disorder risk in adolescents with IBDs may be warranted to enable early identification of vulnerable individuals.
- Integrated, multidisciplinary care addressing both physical and psychological aspects is essential to improve outcomes and quality of life in adolescents with IBDs.

**Abstract:**

**Background/Objectives**: Inflammatory bowel diseases (IBDs) are relapsing–remitting gastrointestinal disorders often emerging in adolescence and frequently associated with psychiatric co-morbidities, including eating disorders (EDs). Deficiency in interoception—awareness of internal bodily sensations—is a transdiagnostic feature in EDs, with emerging evidence suggesting its relevance also in IBDs. This study aimed to assess interoceptive abilities in adolescents with IBDs compared to healthy adolescents. **Methods**: A total of 76 patients with IBDs and 90 healthy controls were enrolled in the study. All participants completed a comprehensive psychometric assessment, including measures of interoceptive sensibility (MAIA-2) and eating-related symptomatology (EDI-3). **Results**: Up to one in six (15.8%) patients with IBDs were found to be at high risk of developing EDs (EDI-3 Eating Disorder Risk Composite scale >70th percentile). Mean MAIA-2 scores were largely comparable, with the exception of the MAIA-2 Trusting subscale, which assesses whether the experience of one’s body is rated as safe and trustworthy. Specifically, patients with IBDs at high risk of developing EDs reported lower scores than both healthy controls and patients with IBDs at low risk of developing EDs, with a statistically significant difference emerging in the comparison with the latter group (*p* = 0.044). **Conclusions**: Adolescents with IBDs who report an elevated risk of developing eating disorders have a psychological profile characterised by increased disordered eating symptomatology, accompanied by selective impairment in interoceptive trust, as evidenced by reduced trust in internal bodily signal. These findings highlight the clinical relevance of thorough clinical assessment and early psychological intervention in this vulnerable population.

## 1. Introduction

Inflammatory bowel diseases (IBDs) are chronic inflammatory disorders of the gastrointestinal tract characterised by a relapsing–remitting course [1]. IBDs include Crohn’s disease (CD), ulcerative colitis (UC), and IBD-type unclassified (IBD-U, where histopathological features at the time of diagnosis do not permit a definitive distinction between CD and UC). The diagnostic process involves a thorough clinical evaluation, biochemical testing, and both endoscopic and histopathological examinations [2]. The aetiology of IBDs is multifactorial, involving genetic, immunological, and environmental factors, and their incidence appears to be on the rise in the paediatric population [3]. In approximately 25% of cases the onset of the disease is during childhood or adolescence [3].

Subjects with IBDs—both children and adolescents—have been reported to experience a higher burden of mental health difficulties. Specifically, there is evidence of an increased prevalence of psychiatric co-morbidities including depression, anxiety disorders, and eating disorders (EDs) [4,5,6,7,8]. Interoception has been defined as “the process by which the nervous system senses, interprets, and integrates signals originating from within the body, thereby providing a moment-to-moment representation of the body’s internal state at both conscious and unconscious levels” [9]. Altered interoceptive processing is recognised as a key feature in EDs [10,11,12] and has been reported in clinical populations with gastrointestinal disorders including IBDs, suggesting a potential shared mechanism underlying disturbances in body–mind communication [13]. Despite initial evidence, to date only a few studies [13,14,15] have investigated interoceptive function in populations with gastrointestinal disorders, particularly in paediatric cohorts. A recent report documented similar interoceptive profiles in adolescents with IBDs at risk of developing EDs and in adolescents with restrictive eating disorders (REDs). These preliminary findings raise the possibility of shared associations underlying both conditions [16]. However, a clear mechanistic account linking chronic gastrointestinal inflammation to alterations in interoceptive processing, which may in turn increase vulnerability to eating disorders, remains insufficiently conceptualised.

To the best of our knowledge, no study has been conducted to explore the possible links between interoceptive function and risk of developing EDs in adolescents with IBDs compared to a non-clinical population. In the present study, we systematically investigated interoceptive awareness in adolescents with IBDs compared with a control group of healthy adolescents. We aimed to determine whether chronic gastrointestinal inflammation is associated with impairments in interoceptive awareness—a function increasingly recognised as playing a central role in both emotional regulation and own body perception.

## 2. Materials and Methods

### 2.1. Study Design and Participants

We recruited a clinical sample of 84 adolescents (aged 13–18 years) from the Pediatric Gastroenterology Unit, Department of Pediatrics, Fondazione IRCCS San Gerardo dei Tintori (Monza, Italy) and the Paediatric Hepatology Gastroenterology and Transplantation, Papa Giovanni XXIII Hospital (Bergamo, Italy), in the period October 2022–April 2024. All patients had a diagnosis of IBDs for at least 6 months. The diagnosis of IBDs—including CD, UC, and IBD-U—was established following the ESPGHAN Revised Porto criteria [1]. We excluded patients with previous formal diagnoses of psychiatric disorders and/or physical co-morbidities (including EDs), intellectual disabilities (IQ < 70), psychosis, and insufficient proficiency in the Italian language. A further eight patients were excluded from the study due to incomplete psychometric data. As a result, a sample of 76 patients (43.4% females) were enrolled: 47 patients with CD, 27 patients with UC, and 2 patients with IBD-U.

Psychometric data from the adolescent IBD group were compared with those of a control group consisting of 90 healthy adolescents aged 13–18 years, recruited between November 2024 and March 2025 from two lower secondary schools and four upper secondary schools located in the northern Italy. Recruitment was carried out on a voluntary basis following approval by the respective school boards, and written informed consent was obtained from all participants’ parents. The healthy control participants were healthy adolescents without any known gastrointestinal or psychiatric conditions prior to inclusion.

Both adolescents and their parents received comprehensive explanations of the study purpose, and written informed consent was obtained from the parents.

### 2.2. Measures

Socio-demographic characteristics, including sex, age at diagnosis and at assessment, and family socio-economic status (evaluated using the Hollingshead index [17]), were collected using a structured ad hoc questionnaire. Clinical information related to the disease, such as duration of illness (in months) and type of IBD, was also systematically recorded.

For participants with IBD, additional disease-specific variables were gathered. These included diagnostic delay, defined as the time interval between the onset of symptoms and formal diagnosis; disease activity at both diagnosis and assessment, evaluated using the Pediatric Crohn’s Disease Activity Index (PCDAI) for Crohn’s disease [18] and the Pediatric Ulcerative Colitis Activity Index (PUCAI) for ulcerative colitis [19]; current pharmacological treatment; total number of disease relapses (overall and within the previous six months); number of corticosteroid treatment cycles (overall and in the last six months); and the number of hospitalizations.

In addition to providing socio-demographic and clinical data, all participants completed a self-report psychometric battery, which included the Italian versions of validated instruments assessing interoceptive sensibility [20] and ED symptomatology [21]:

The Multidimensional Assessment of Interoceptive Awareness, Version 2 (MAIA-2), is a self-report instrument designed to evaluate multiple facets of interoception. It comprises 37 items rated on a six-point Likert scale [20], with higher scores reflecting greater self-perceived interoceptive awareness. The questionnaire is organised into eight subscales: Noticing (awareness of uncomfortable, comfortable and neutral body sensations’), Not-Distracting (‘tendency not to ignore or distract oneself from sensations of pain or discomfort’), Not-Worrying (‘tendency not to worry or experience emotional distress with sensations of pain or discom-fort’), Attention Regulation (‘ability to sustain and control attention to body sensations’), Emotional Awareness (‘awareness of the connection between body sensations and emotional states’), Self-Regulation (‘ability to regulate distress by attention to body sensations’), Body Listening (‘active listening to the body for in-sight’) and Trusting (‘experience of one’s body as safe and trustworthy’). Cronbach’s alpha values for the eight subscales range from 0.64 to 0.83.

The Eating Disorder Inventory-3 (EDI-3) is a self-administered instrument used to evaluate psychological features associated with eating disorders. It consists of 91 items organized into 12 primary scales. Three of these specifically assess core eating disorder symptoms (Drive for Thinness, Bulimia, and Body Dissatisfaction), while the other nine explore broader psychological dimensions linked to disordered eating, including Low Self-Esteem, Personal and Interpersonal Alienation, Interpersonal Insecurity, Interoceptive Deficits, Emotional Dysregulation, Perfectionism, Ascetism, and Maturity Fears. Based on these primary scales, six composite indices can be derived: one reflecting eating disorder risk (Eating Disorder Risk Composite, EDRC) and five summarizing key psychological domains (Ineffectiveness, Interpersonal Problems, Affective Problems, Overcontrol, and Global Psychological Maladjustment). The Italian adaptation of the EDI-3 has shown good psychometric properties, with internal consistency coefficients ranging between 0.80 and 0.90, and excellent test–retest reliability, with values between 0.93 and 0.98 [21].

For the purpose of this study, the IBD sample was divided into two groups based on the risk of developing EDs, as determined by the EDI-3 EDRC cut-off score: participants scoring above 70th percentile were classified as high risk (IBD-HR), while those scoring below 70th percentile were considered low risk (IBD-LR).

### 2.3. Statistical Analysis

Since data were not normally distributed, continuous variables were presented as means and standard deviations, as well as medians and interquartile ranges (IQR), whereas categorical variables were presented as absolute or percent frequencies. The Chi-square test (or Fisher’s exact test for frequencies below 5) was used to compare categorical variables between groups.

Patients with IBDs who scored above the 70th percentile on the EDI-3 EDRC subscale (cut-off value for pathological scores) were compared with individuals who scored below the cut-off and with HCs. All comparisons of socio-demographic variables among the three groups (IBD-HR vs. IBD-LR vs. HCs) were carried out through univariate analysis using the non-parametric Kruskal–Wallis test for multiple comparisons. Comparisons of the clinical characteristics between the two IBD groups were carried out through univariate analysis using the non-parametric Kruskal–Wallis test for multiple comparisons. Since the three groups differed for age at evaluation, gender, and socio-economic status, a non-parametric ANCOVA with these variables as covariates was used to compare the three groups for EDI-3 and MAIA-2 scores. Finally, a multivariate model of logistic regression analyses was conducted with IBD-EDRC > 70 as the dependent variable and MAIA-2 scores and covariates as independent variables. Values of *p* ≤ 0.05 were considered to be statistically significant. All statistical analyses were performed using the Statistical Package for the Social Sciences (SPSS, version 29.0.1.0; IBM Corp., Armonk, NY, USA) [6].

## 3. Results

We analysed data from a total of 166 adolescents aged 13–18 years: 76 adolescents with IBDs and 90 HCs. The socio-demographic and clinical characteristics of the two groups are summarised in Table 1. Patients with IBDs and HCs were comparable in both age and gender, whereas they differed in socio-economic status (*p* < 0.001).

Among the 76 adolescents with IBDs, 12 (15.8%; 7 with UC and 5 with CD) were identified as being at risk of developing EDs (IBD-HR), as they scored above the recommended cut-off (>70th percentile) on the EDI-3 EDRC subscale.

The comparison of socio-demographic characteristics among the three groups (IBD-HR, IBD-LR, and HCs) is shown in Table 2. Females represented 75.0% of subjects in the IBD-HR group, 37.5% in IBD-LR, and 54.4% in HCs (IBD-HR vs. IBD-LR: *p* = 0.025; IBD-LR vs. HCs: *p* = 0.049; IBD-HR vs. HCs: *p* = 0.224). Socio-economic status (SES) was higher in HCs, with a median score of 42.0, compared to a median of 32.0 in IBD-HR and 29.0 in IBD-LR. A statistically significant difference in SES was found between HCs and both IBD groups (IBD-HR vs. IBD-LR: *p* = 0.934; IBD-LR vs. HCs: *p* = 0.001; IBD-HR vs. HCs: *p* = 0.003).

With regard to age at the time of questionnaire completion, a higher median age was observed in the patients with IBDs, with a median age of 16.0 years in the IBD-HR group, 15.5 years in IBD-LR, and 15.0 years in HCs. A statistically significant difference in age was observed between the IBD-HR group and the HCs group (IBD-HR vs. IBD-LR: *p* = 0.076; IBD-LR vs. HCs: *p* = 0.207; IBD-HR vs. HCs: *p* = 0.013).

The disease characteristics of patients with IBDs at risk of developing EDs and those without risk of developing EDs are reported in Table 3.

Clinical and disease-related characteristics did not differ significantly between the IBD-HR group and the IBD-LR group. Likewise, the distribution of IBD phenotypes (CD, UC, IBD-U) was similar across groups (*p* = 0.618). Clinical disease activity at diagnosis and at the time of evaluation showed no significant differences between the two groups (*p* = 0.438 and 0.778, respectively). Mean diagnostic delay, pharmacological therapy (including use of biological drugs and polytherapy), number of steroid cycles, number of relapses, and hospital admissions were also comparable between the two groups (all *p* > 0.05).

Comparisons of the EDI-3 scores among the three groups are summarised in Table 4. The IBD-HR group consistently reported the highest median scores across EDI-3 subscales, frequently exceeding clinical cut-offs, whereas the IBD-LR group showed intermediate values and HCs reported predominantly non-clinical scores. Patients in the IBD-HR group showed significantly higher scores than those in the IBD-LR group across most EDI-3 subscales, except for Interpersonal Alienation (*p* = 0.135) and Maturity Fears (*p* = 0.209). Comparisons between IBD-HR and HCs revealed significant differences for Drive for Thinness (*p* < 0.001), Bulimia (*p* = 0.023), Body Dissatisfaction (*p* < 0.001), Low Self-Esteem (*p* = 0.003), Personal Alienation (*p* = 0.002), Interoceptive Deficits (*p* = 0.005), Emotional Dysregulation (*p* = 0.014), Perfectionism (*p* = 0.012), Asceticism (*p* = 0.003), Impulse Regulation (*p* < 0.001), Interpersonal Problems Composite (*p* = 0.002), Overcontrol Composite (*p* = 0.004), and Global Psychological Maladjustment Composite (*p* = 0.001). Differences were not significant for Interpersonal Insecurity (*p* = 0.073), Interpersonal Alienation (*p* = 0.161), Maturity Fears (*p* = 0.592), or Affective Problems Composite (*p* = 0.066). When comparing IBD-LR and HCs, significant differences emerged for Drive for Thinness (*p* = 0.040), Bulimia (*p* = 0.020), Impulse Regulation (*p* = 0.032), Perfectionism (*p* = 0.006), Asceticism (*p* = 0.017), and Global Psychological Maladjustment Composite (*p* = 0.002), whereas no significant differences were observed across the remaining EDI-3 subscales.

Comparisons of the MAIA-2 scores are reported in Table 5. Overall, the IBD-HR group showed generally lower scores across most MAIA-2 subscales compared with the other two groups, although differences were limited in scope. A statistically significant difference between groups emerged for the Trusting subscale only. Specifically, the IBD-LR group reported significantly higher Trusting scores than the IBD-HR group (*p* = 0.044), suggesting greater confidence in bodily sensations and a stronger perception of the body as safe and reliable. No significant differences were observed between HCs and the IBD groups on this subscale (*p* > 0.05). There were no statistically significant differences among the three groups for the Noticing, Not Distracting, Attention Regulation, Emotional Awareness, Self-Regulation, or Body Listening subscales. A trend towards significance was observed for the Not Worrying subscale, with the IBD-LR group reporting higher scores than the IBD-HR group (*p* = 0.095), although this difference did not reach statistical significance. Across all analyses, the most consistent and robust finding was a reduction in the MAIA-2 Trusting dimension among IBD-HR patients, suggesting a selective interoceptive vulnerability rather than a global interoceptive deficit.

As reported in Table 6, the MAIA-2 subscales and covariates (socio-economic status, age at assessment and female gender) were included in a logistic regression model using IBD-EDRC > 70 as the dependent variable. The model was statistically valid (B = −0.374, *p* = 0.036). Higher socio-economic status (*p* = 0.018) and female gender (*p* = 0.039) were identified as significant predictors for the risk of developing EDs in individuals with IBDs.

## 4. Discussion

Impairments in interoceptive processing are recognised as a core feature of EDs [10,11,12] and appear to be implicated in the pathophysiology of gastrointestinal disorders, including IBDs [13]. In our sample, up to one in six (15.8%) adolescents with IBDs screened positive for the risk of developing EDs (IBD-HR), based on their scores above the pathological cut-off (>70th percentile) on the EDI-3 EDRC. This prevalence figure is approximately six times higher than the 2.7% risk reported in the general adolescent population [22]. Moreover, it aligns with previous studies indicating that the risk of developing EDs in patients with IBDs ranges from 5% to 17%, depending on age and IBD phenotype, thus supporting a strong association between IBDs and EDs [5,23].

Our IBD sample was divided into two groups based on EDI-3 EDRC scores: low risk of developing EDs (IBD-LR; n = 64, 84.2%) and high risk (IBD-HR; n = 12, 15.8%). Both groups were compared to a HCs group. The groups were not fully matched in terms of age, gender, and socio-economic status, with a marked female predominance in the IBD-HR subgroup (75.0%), compared to 54.4% among HCs and 37.5% in the IBD-LR group. This gender distribution is consistent with previous data highlighting a higher prevalence of ED symptoms among female adolescents, particularly body dissatisfaction, drive for thinness, binge eating, and compensatory behaviours [24].

Comparison between the two IBD groups revealed significantly higher EDI-3 scores in the IBD-HR group across all subscales except Maturity Fear and Interpersonal Alienation. This suggests a heightened risk of developing EDs in this group and confirming the utility of EDI-3 EDRC subscores as a marker of vulnerability. Our results are consistent with the findings of a previous study by Cordero et al. [25], who reported a correlation between elevated EDI-3 EDRC subscores and increased risk of EDs in a sample of Latina women. Furthermore, when compared with healthy adolescents, the IBD-HR group demonstrated a trend towards higher EDI-3 scores, further suggesting increased susceptibility to EDs.

Analysis of MAIA-2 ratings revealed largely comparable scores across groups, with a significant difference in the Trusting dimension only. Adolescents in the IBD-HR group reported lower Trusting scores than both the IBD-LR group and HCs, with the difference between IBD-HR and IBD-LR reaching statistical significance. Moreover, higher socio-economic status and female gender predicted the risk of developing an ED in the IBDs group. These results suggest reduced confidence in own body sensations and diminished perception of the body as safe and reliable in patients with IBDs who are at risk of developing EDs. This is consistent with previous research identifying compromised interoceptive trust as a vulnerability factor in EDs, particularly anorexia nervosa. In a previous study by Phillipou et al. [26], patients with anorexia nervosa reported significantly lower scores on the MAIA Trusting subscale compared to HCs, raising the possibility that pathological conditions exacerbate difficulties in perceiving the body as a safe and trustworthy entity. Similarly, Brown et al. [27] confirmed the validity of the MAIA-2 scale in adolescents and adults with EDs, showing that low Trusting scores are associated with increased dietary restriction, body image concerns, and disordered eating behaviours. Additional studies have shown that perceiving the body as unsafe mediates the relationship between interoceptive dysfunction and ED symptoms [28] and that deficit in interoception in adolescents with EDs increase the risk of suicide attempts and suicide ideation [29]. Our findings thus support the relevance of reduced interoceptive trust in adolescents with IBDs at high risk of developing EDs.

Our study has several limitations. First, the relatively small sample size may reduce statistical power and limit the generalisability of findings. In particular, the small size of the IBD-HR group (n = 12) limits statistical robustness and increases the risk of type II error. Second, psychological measures were obtained via self-report questionnaires, which may introduce response biases. In particular, social desirability and/or the tendency to provide less truthful responses may affect the validity of the collected data. It is also important to note that participants were classified as high or low risk using the EDI-3 Eating Disorder Risk Composite. Consequently, some of the differences observed across the EDI-3 subscales may reflect this classification rather than entirely independent psychological differences. Third, HCs were recruited within a later time window than the clinical cohort, which may introduce potential temporal or contextual bias. This should be considered when interpreting the findings. Moreover, referral bias must be considered: HCs were recruited from volunteers across schools in northern Italy, potentially introducing selection bias related to socio-demographic or psychological characteristics. These include higher motivation or socio-economic status, which may further limit the representativeness of our sample. Finally, residual confounding due to differences in recruitment sources (school-based controls versus hospital-based clinical sample) cannot be excluded, despite statistical adjustment for socio-demographic variables.

## 5. Conclusions

IBDs are disabling conditions that have a significant impact on the patient’s quality of life, with repercussions also on psychological and emotional levels. This is particularly relevant during adolescence, a crucial phase for the consolidation of one’s identity and personality, as well as a fundamental period for the development of interpersonal relationships.

Our data suggest the existence of a subpopulation of adolescents with IBDs experiencing greater psychological distress, where interoceptive awareness and emotional functioning display features that are similar to those commonly observed in individuals diagnosed with EDs. This similarity may reflect potential associations between the two conditions. Our findings highlight the need for closer monitoring and implementation of targeted interventions aimed at preventing and promptly addressing potential psychiatric comorbidities in at-risk clinical subgroups.

## Figures and Tables

**Table 1:** Comparison of socio-demographical and clinical characteristics of patients with IBDs and HCs.

	IBDs (N = 76)		HCs (N = 90)		*p*-Value
Female, N (%)	33 (43.4)		49 (54.4)		0.165
Age at evaluation, mean (s.d.); median (IQR)	15.51 (1.88)	16.00 (3.00)	15.09 (1.43)	15.00 (2.00)	0.059
SES, mean (s.d.); median (IQR)	31.41 (12.57)	29.00 (21.00)	43.19 (8.82)	42.00 (13.60)	<0.001 **
Disease duration (months), mean (s.d.); median (IQR)	43.91 (35.76)	33.00 (41.00)	-		-

Abbreviations: IBDs, Inflammatory Bowel Diseases; HCs, healthy controls, SES, socio-economic status. IQR, interquartile range. ** *p* ≤ 0.001.

**Table 2:** Comparison of socio-demographic characteristics across adolescents with IBD at high risk of developing EDs (IBD-HR), adolescents with IBD at low risk of developing EDs (IBD-LR), and healthy controls (HCs).

	IBD-HR (N = 12)		IBD-LR (N = 64)		HCs (N = 90)		IBD-HR vs. IBD-LR *p*	IBD-LR vs. HCs *p*	IBD-HR vs. HCs *p*
Female gender, N (%)	9 (75.0)		24 (37.5)		49 (54.4)		0.025 *	0.049 *	0.224
SES, mean (s.d.); median (IQR)	31.63 (12.51)	32.00 (21.00)	31.37 (12.67)	29.00 (21.80)	43.19 (8.82)	42.00 (13.60)	0.934	<0.001 *	0.003 *
Age at questionnaire, mean (s.d.); median (IQR)	16.33 (1.56)	16.00 (3.00)	15.36 (1.90)	16.00 (3.00)	15.09 (1.43)	15.00 (2.00)	0.076	0.207	0.013 *

Abbreviations: IBD, Inflammatory Bowel Disease; HCs, healthy controls; SES, socio-economic status; IQR, interquartile range. * *p* ≤ 0.05.

**Table 3:** Clinical and disease-related characteristics of the IBD sample, stratified by eating disorder risk (N = 76).

	IBD-HR (N = 12)	IBD-LR (N = 64)	*p*
Diagnosis, N (%)			0.618
CD	5 (40.0)	22 (34.4)	
UC	6 (50.0)	41 (64.1)	
IBD-U	1 (10.0)	1 (1.5)	
Clinical Disease Activity at diagnosis, N (%)			
remission	2 (16.7)	4 (6.3)	
mild	5 (41.7)	26 (40.6)	0.438
moderate-severe	5 (41.7)	34 (53.1)	
Clinical Disease Activity at evaluation, N (%)			
remission	10 (83.3)	49 (59.8)	
mild	2 (16.7)	13 (15.9)	0.778
moderate-severe	0 (0.0)	2 (2.4)	
Diagnostic delay (months), mean (s.d.)	6.17 (6.76)	6.41 (7.33)	0.748
Pharmacological therapy, N (%)			
Biological drugs	7 (58.3)	39 (60.9)	0.554
Polytherapy	6 (50.0)	24 (37.5)	0.524
Number of steroid cycles, mean (s.d.)	1.42 (1.62)	1.05 (1.29)	0.451
Number of steroid cycles in the previous six months, mean (s.d.)	0.17 (0.39)	0.17 (0.38)	0.965
Number of relapses, mean (s.d.)	1.50 (1.62)	1.25 (1.68)	0.513
Number of relapses in the previous six months, mean (s.d.)	0.08 (0.29)	0.28 (0.72)	0.366
Number of hospitalisations, mean (s.d.)	0.67 (0.88)	0.98 (1.13)	0.386

Abbreviations: UC, ulcerative colitis; CD, Crohn’s disease; IBD, Inflammatory Bowel Disease; IBD-U, unclassified Inflammatory Bowel Disease.

**Table 4:** Comparison of EDI-3 scores across adolescents with IBD at high risk of developing EDs (IBD-HR), adolescents with IBD at low risk of developing EDs (IBD-LR), and healthy controls (HCs), adjusted for age at evaluation, gender, and socio-economic status.

	IBD-HR		IBD-LR		HCs		IBD-HR vs. IBD-LR	IBD-LR vs. HCs	IBD-HR vs. HCs
	Mean (s.d.)	Median (IQR)	Mean (s.d.)	Median (IQR)	Mean (s.d.)	Median (IQR)	*p*-Value	*p*-Value	*p*-Value
EDI-DT	78.42 (6.64)	73.00 (12.00)	26.70 (24.35)	34.00 (45.00)	39.17 (31.55)	34.00 (53.00)	<0.001 **	0.040 *	<0.001 **
EDI-B	77.92 (18.43)	61.00 (18.00)	37.20 (30.75)	42.00 (69.00)	48.47 (31.93)	52.00 (45.00)	0.001 *	0.020 *	0.023 *
EDI-BD	81.92 (8.87)	64.00 (13.00)	25.25 (18.16)	29.00 (21.00)	34.74 (30.09)	26.00 (46.00)	<0.001 **	0.142	<0.001 **
EDI-LSE	75.50 (14.32)	67.00 (19.00)	36.75 (25.38)	47.00 (35.00)	44.14 (29.42)	46.50 (53.00)	<0.001 **	0.098	0.003 *
EDI-PA	79.25 (14.16)	65.00 (20.00)	38.39 (25.93)	44.00 (43.00)	44.49 (31.19)	44.00 (60.00)	<0.001 **	0.192	0.002 *
EDI-II	72.92 (20.05)	73.00 (33.00)	50.64 (24.01)	60.00 (33.00)	53.48 (26.78)	52.00 (40.00)	0.045 *	0.605	0.073
EDI-IA	62.00 (26.87)	59.00 (50.00)	41.72 (26.56)	45.00 (47.00)	42.62 (29.88)	36.00 (54.00)	0.135	0.799	0.161
EDI-ID	84.75 (13.79)	78.00 (18.00)	44.67 (26.42)	51.00 (46.00)	51.89 (31.94)	58.00 (56.00)	<0.001 **	0.060	0.005 *
EDI-ED	78.08 (27.27)	64.00 (22.00)	42.17 (29.78)	50.00 (53.00)	48.68 (31.23)	43.00 (55.00)	0.004 *	0.330	0.014 *
EDI-P	75.67 (27.24)	59.00 (27.00)	39.36 (29.36)	41.00 (51.00)	56.06 (30.94)	59.00 (60.00)	<0.001 **	0.006 *	0.012 *
EDI-A	78.58 (11.97)	69.00 (21.00)	43.20 (25.80)	50.00 (36.00)	51.57 (26.80)	57.00 (44.00)	<0.001 **	0.017 *	0.003 *
EDI-MF	60.00 (24.33)	59.00 (36.00)	47.94 (26.35)	53.00 (38.00)	54.13 (26.26)	56.00 (38.00)	0.209	0.146	0.592
EDI-IC	83.17 (5.72)	69.00 (10.00)	28.34 (20.20)	50.00 (42.00)	39.48 (30.65)	50.00 (53.00)	<0.001 **	0.032 *	<0.001 **
EDI-IPC	79.00 (12.22)	70.00 (19.00)	38.42 (24.54)	57.00 (38.00)	45.42 (29.85)	50.00 (46.00)	<0.001 **	0.139	0.002 *
EDI-APC	71.17 (20.46)	74.00 (24.00)	48.02 (24.68)	52.00 (47.00)	49.93 (27.82)	54.00 (54.00)	0.048 *	0.707	0.066
EDI-OC	84.58 (15.54)	65.00 (30.00)	44.27 (27.19)	46.00 (39.00)	51.96 (30.86)	63.00 (43.00)	<0.001 **	0.086	0.004 *
EDI-GPMC	81.58 (13.87)	73.00 (20.00)	41.13 (26.23)	58.00 (33.00)	56.97 (26.98)	58.00 (46.00)	<0.001 **	0.002 **	0.001 *

Abbreviations: IBD, Inflammatory Bowel Disease; HCs, healthy controls; DT, Drive for Thinness; B, Bulimia; BD, Body Dissatisfaction; LSE, Low Self Esteem; PA, Personal Alienation; II, Interpersonal Insecurity; IA, Interpersonal alienation; ID, Interoceptive Deficits; ED, Emotional Dysregulation; P, Perfectionism; A, Ascetism; MF, Maturity Fear; IC, Ineffectiveness Composite; IPC, Interpersonal Problems; APC, Affective Problems Composite; OC, Overcontrol Composite; GPMC, Global Psychological Maladjustment Composite; IQR, interquartile range. * *p* < 0.05, ** *p* ≤ 0.001.

**Table 5:** Comparison of MAIA-2 scores across adolescents with IBD at high risk of developing EDs (IBD-HR), adolescents with IBD at low risk of developing EDs (IBD-LR), and healthy controls (HCs), adjusted for age at evaluation, gender, and socio-economic status.

MAIA-2 Subscales	IBD-HR		IBD-LR		HCs		ANCOVA IBD-HR vs. IBD-LR	ANCOVA IBD-LR vs. HCs	ANCOVA IBD-HR vs. HCs
	Mean (s.d.)	Median (IQR)	Mean (s.d.)	Median (IQR)	Mean (s.d.)	Median (IQR)	*p*	*p*	*p*
Noticing	2.87 (1.05)	2.75 (1.50)	2.27 (1.38)	2.62 (2.56)	2.64 (1.02)	2.50 (1.31)	0.814	0.425	0.860
Not Distracting	2.15 (1.14)	1.83 (1.33)	2.44 (0.99)	2.50 (1.00)	2.21 (0.83)	2.17 (0.83)	0.352	0.362	0.631
Not Worrying	2.17 (1.10)	2.40 (1.80)	2.92 (1.02)	2.90 (1.20)	2.57 (1.09)	2.50 (1.60)	0.095	0.076	0.429
Attention Regulation	2.37 (0.86)	2.57 (1.86)	2.63 (1.11)	2.57 (1.36)	2.59 (0.93)	2.57 (1.18)	0.417	0.420	0.679
Emotional Awareness	3.07 (1.25)	3.80 (2.60)	2.85 (1.26)	3.00 (1.60)	2.83 (1.15)	3.10 (1.20)	0.408	0.607	0.264
Self-Regulation	1.87 (1.11)	2.25 (2.25)	2.41 (1.21)	2.50 (1.75)	2.30 (1.08)	2.25 (1.50)	0.397	0.351	0.700
Body Listening	1.86 (1.03)	2.00 (1.67)	2.34 (1.25)	2.50 (2.00)	2.13 (1.10)	2.00 (1.67)	0.255	0.226	0.589
Trusting	2.17 (0.96)	2.67 (1.33)	3.38 (1.18)	3.50 (1.41)	2.98 (1.40)	3.00 (2.33)	0.044 *	0.056	0.280

Abbreviations: IBD, Inflammatory Bowel Disease; HCs, healthy controls; *p*, *p*-value; IQR, interquartile range. * *p* < 0.05.

**Table 6:** Multivariate model with MAIA-2 subscales, age, SES and female gender as predictors of the risk of developing eating disorders in patients with IBD at high risk of developing EDs (IBD-HR).

				95% C.I. for EXP(B)	
Predictive Variables	B	*p* Value	OR	Inf	Sup
Noticing	0.392	0.070	1.481	0.969	2.263
Not Distracting	−0.384	0.111	0.681	0.425	1.092
Not Worrying	−0.232	0.325	0.793	0.500	1.258
Attention Regulation	0.233	0.459	1.262	0.682	2.339
Emotional Awareness	−0.130	0.581	0.878	0.554	1.393
Self-Regulation	0.087	0.737	1.090	0.658	1.806
Body Listening	−0.535	0.045	0.586	0.347	0.988
Trusting	−0.001	0.996	0.999	0.688	1.450
Socio-economic status (SES)	0.041	0.018 *	1.042	1.007	1.077
Age at questionnaire	−0.153	0.220	0.858	0.673	1.096
Female gender	−0.895	0.039 *	0.409	0.175	0.957
	R^2^ 0.204				

Abbreviations: OR, odds ratio. * *p* < 0.05.

## Data Availability

The datasets used and analysed in the current study are available from the corresponding author on reasonable request due to privacy.
